# Gathering Opinions on Depression Information Needs and Preferences: Samples and Opinions in Clinic Versus Web-Based Surveys

**DOI:** 10.2196/mental.7231

**Published:** 2017-04-24

**Authors:** Matthew T Bernstein, John R Walker, Kathryn A Sexton, Alan Katz, Brooke E Beatie, Mobilizing Minds Research Group

**Affiliations:** ^1^ Faculty of Arts Department of Psychology University of Manitoba Winnipeg, MB Canada; ^2^ Faculty of Health Sciences Department of Clinical Health Psychology University of Manitoba Winnipeg, MB Canada; ^3^ Manitoba Centre for Health Policy Department of Community Health Sciences University of Manitoba Winnipeg, MB Canada

**Keywords:** depression, psychotherapy, drug therapy, Internet, survey methodology

## Abstract

**Background:**

There has been limited research on the information needs and preferences of the public concerning treatment for depression. Very little research is available comparing samples and opinions when recruitment for surveys is done over the Web as opposed to a personal invitation to complete a paper survey.

**Objective:**

This study aimed to (1) to explore information needs and preferences among members of the public and (2) compare Clinic and Web samples on sample characteristics and survey findings.

**Methods:**

Web survey participants were recruited with a notice on three self-help association websites (N=280). Clinic survey participants were recruited by a research assistant in the waiting rooms of a family medicine clinic and a walk-in medical clinic (N=238) and completed a paper version of the survey.

**Results:**

The Clinic and Web samples were similar in age (39.0 years, SD 13.9 vs 40.2 years, SD 12.5, respectively), education, and proportion in full time employment. The Clinic sample was more diverse in demographic characteristics and closer to the demographic characteristics of the region (Winnipeg, Canada) with a higher proportion of males (102/238 [42.9%] vs 45/280 [16.1%]) and nonwhites (Aboriginal, Asian, and black) (69/238 [29.0%] vs 39/280 [13.9%]). The Web sample reported a higher level of emotional distress and had more previous psychological (224/280 [80.0%] vs 83/238 [34.9%]) and pharmacological (202/280 [72.1%] vs 57/238 [23.9%]) treatment. In terms of opinions, most respondents in both settings saw information on a wide range of topics around depression treatment as very important including information about treatment choices, effectiveness of treatment, how long it takes treatment to work, how long treatment continues, what happens when treatment stops, advantages and disadvantages of treatments, and potential side effects. Females, respondents with a white background, and those who had received or felt they would have benefited from therapy in the past saw more information topics as very important. Those who had received or thought they would have benefited in the past from medication treatment saw fewer topics as important. Participants in both groups expressed an interest in receiving information through discussion with a counselor or a physician, through written brochures, or through a recommended website.

**Conclusions:**

The recruitment strategies were helpful in obtaining opinions from members of the public with different concerns and perspectives, and the results from the two methods were complementary. Persons coping with emotional distress and individuals not specifically seeking help for depression would be interested in information to answer a wide range of important questions about depression treatment. The Clinic sample yielded more cultural diversity that is a closer match to the population. The Web sample was less costly to recruit and included persons who were most interested in receiving information.

## Introduction

### Importance of Health Information

Major depression is one of the most common and disabling mental health problems in the community [1]. It is important to understand how persons with depression prefer to receive information about treatment and what they want to know about treatment options. The exchange of information about treatment options is essential in shared decision-making and obtaining informed consent for treatment [2]. There are large differences among people in the amount of information they wish to receive concerning treatment options and how they prefer to receive this information [3]. In a wide range of health conditions, information needs early in the course of treatment may differ from information needs later in the course of treatment, or when considering changes in treatment [4]. Often even those who are well connected with health services have information needs that have not been addressed in the course of regular clinical contacts [5]. It is helpful to have information resources available that are flexible enough to allow for differing information needs and that are low enough in cost to be easily accessible to patients receiving health care and to those searching for health information on the Web [3,4].

Health information preferences are influenced by attitudinal and motivational factors [6]. The theory of planned behavior [7,8], for example, suggests that the approach people take to health information seeking will be influenced by anticipated benefits (attitudes), the influence of important individuals (subjective norms), confidence in one’s ability to use information (perceived behavioral control), and the degree to which the person intends to actually seek information (intent) [3]. Given this context, it is helpful to explore what information people consider to be important in decision making around treatments for depression (attitudes), whom people consider turning to for help in making health decisions (subjective norms), and their views on preferred ways to receive information (perceived behavioral control).

Whereas there is a great deal of health information on the Web, and the public increasingly uses the Web to access health information [9], there are questions about the quality and the comprehensiveness of the information available [10]. Previous research suggests that currently available information does not address many of the important questions that patients have about managing health [11] and mental health problems [12]. Current information on the Web often focuses on a description of the health problem with a description of the treatment options with little evaluative information based on research evidence [13,14]. In a recent systematic review of the information and decision-making needs of people with mental disorders [15], only 12 studies were identified with 6 addressing depression. The results suggest that much more research is needed in this area. One study [16] found that many patients received very limited information when making treatment choices and most desired more information.

### Paper- and Web-Based Survey Methods

Obtaining the opinions of those who may be interested in specific types of health information is challenging in survey research. The most favorable research situation [17] is when the researchers can clearly establish the survey population (the group they wish to generalize results to—in this case people wanting information about depression and its treatment), the sampling frame (a list of possible participants from which a sample is to be drawn), and a random sample from this sampling frame. Whereas in some cases, this information is available (such as national health and social surveys which obtain a representative sample from a specific geographic area), in many situations there is not a good source of information to provide a list of potential survey participants. In these situations, it is often necessary for researchers to consider opportunity or convenience samples that may not be clearly representative of a larger population but may provide helpful information about a research question in any case. We identified two approaches to obtaining survey samples to consider the information needs of persons who are currently or may in the future be seeking information about treatments for depression. The first was to sample persons visiting a primary care medical setting for routine care. When people decide to seek treatment for depression, this is often where they first go, and many people receive treatment for depression exclusively in a primary care setting [18]. People with chronic medical conditions are also at greater risk for the development of mood disorders [19]. A second approach was to post a notice about the survey on the websites of organizations focused on informing the public about mental health problems. Members of the public frequently search for information about health problems and treatment on the Web [9].

With the increased use of the Web in the last 20 years, Web-survey research has become very prevalent. There has been limited research comparing the results when recruitment is done over the Web (responding to a mass mailing or clicking on a link in a posted invitation) as opposed to being done with a personal invitation to complete a paper survey in a community setting [20]. Much of the research in this area has focused on comparing the measurement properties of established measures administered in Web-based and paper-based formats [21-24]. The comparability of Web-based and paper-based surveys has been assessed in a variety of different situations and generally, this research has found that responses are reasonably similar between paper and Web-based questions and structured measures, especially when demographic factors are considered [20,25].

One advantage of traditional survey administration is that it is possible to determine response rate. Whether the survey is administered via mail or email invitations to specific persons or when participants are approached in a medical clinic waiting room in person, researchers are able to determine how many persons responded out of the total number invited to participate. It is also possible to obtain more information about the representativeness of respondents as compared with nonrespondents when there is a sampling frame with detailed information about those invited to participate. In surveys carried out in public areas (such as a medical waiting room), it is possible to gather information about respondents but no information is available on the characteristic of nonrespondents. For both the medical clinic survey and the Web survey, it is possible to compare the characteristics of respondents with characteristics of persons in the region.

Web survey recruitment and administration, on the other hand, has the advantage of lower cost and of reaching a broader audience, that is, people that differ demographically and geographically [26]. Another advantage is convenience; there are few restrictions on the time and place participants can access the survey, as long as they have access to the Web. Web-based surveys can be constructed to minimize the number of missed questions and to easily branch into different questions based on earlier answers.

### Aims of This Study

Professionals commonly produce resources for the public with limited knowledge of what information is of interest to consumers and the public at large. Hence, there remains a need to understand the information needs and preferences of the public concerning treatment choices for depression. The first aim was to explore the following questions using two different survey approaches: (1) What information would be important to members of the public? (2) To whom would they turn for advice? (3) How would they prefer to receive information? and (4) What treatment services would they see as most helpful if they were experiencing problems with depression?

The second aim of this study was to compare respondent characteristics and information needs and preferences between participants recruited in a clinical setting for paper surveys and those recruited on the Web through self-help organization websites.

## Methods

This study was approved by the University of Manitoba Research Ethics Board (REB).

### Participants

#### Clinic Survey

This survey was conducted in two medical clinics in Winnipeg, Canada. One was a large clinic near a teaching hospital where patients had scheduled appointments with family physicians. The second clinic, near a large shopping center, provided both walk-in services and a limited number of scheduled appointments with family physicians. Under Canada’s publicly funded health care system, there is no charge for physician visits. Of 340 patients in the waiting rooms invited to complete the survey, 241 agreed to participate and 231 were included in the analyses (67.9% of the total).

#### Web Survey

Persons visiting the websites of the Anxiety Disorders Association of Manitoba, the Canadian Mental Health Association (Winnipeg Region), and the Mood Disorders Association of Manitoba were invited to participate in the survey by a notice posted on each of these websites (see Multimedia Appendix 1 for recruitment notice). These websites are widely visited by members of the public searching for information about common mental health problems. We made the survey available on the Anxiety Disorders Association of Manitoba website because persons with anxiety problems have a higher rate of problems with major depression than the public in general. Website visitors who were interested in participating could click on a link to the survey, which was presented through a web-based survey tool called SurveyGizmo (SurveyGizmo, 4888 Pearl East Cir. Suite 100, Boulder, Colorado). Once the link was clicked, participants viewed the information and informed consent page.

### Procedure

#### Clinic Survey

A research assistant invited persons waiting for appointments to participate. Those who provided consent completed the 20-min anonymous survey in the waiting room. An honorarium (a gift card, Can $5 value) was provided.

#### Web Survey

The same measures were used in the Web survey as in the Clinic survey. After the participants provided their consent, they were presented with the survey. Enrollment continued for approximately 2 months without providing compensation to participants. After 2 months of data collection, in order to increase participation, we provided a $10 gift card to a grocery or coffee retailer as compensation for participation. Whereas 280 participants answered the demographic questions, approximately 262 individuals answered the information needs and preferences questions. Just over half (54.3%) of this group of respondents received an honorarium for participating in the study. The survey duration was approximately 20 min.

### Measurement

In developing questions, we considered what information might be important to a well-informed person making treatment decisions. The questions considered the logical sequence of events in decisions about treatment: treatment choices; the characteristics of each treatment; treatment cost, effectiveness, and duration; what happens when the treatment is stopped; and the risks of treatment. Draft questions were reviewed in a consensus meeting involving members of a self-help anxiety association, psychiatrists and psychologists specializing in the treatment of anxiety, and a family physician from a teaching clinic. There was a high degree of consensus on the final questions. These questions have been used in previous research on information needs of young adults [27], parents of anxious children [28], and persons with inflammatory bowel disease [29] and have provided consistent findings in these different contexts. The topic areas were consistent with themes developed in focus groups with young adults (unpublished data). The complete survey is shown in Multimedia Appendix 2.

#### Sociodemographic Information

Participants provided information concerning their gender, age, marital status, education level, main activity (employment), and cultural or ethnic background.

#### Information Preferences

To set the context, respondents read a vignette describing a person with depression matching their gender. The vignette was brief (7 lines) and described a person with significant depression meeting five of the nine Diagnostic and Statistical Manual of Mental Disorders-5 (DSM-5) criteria for major depression [30] with no mention of suicidal thoughts. Next, they were asked whether or not they had used the Web to search for health care information and how familiar they are with the types of help available for depression. Then they received the instructions: “At some time in your life you, a close friend, or a close family member might be having a problem with depression. What information would be important to you in considering the kinds of help available for depression?” These instructions were followed by 27 questions focusing on content areas of information that they might consider important in making decisions.

Then participants were asked 5 questions about the medium they would prefer in receiving information. They were also asked, “How likely would you be to talk to one of the following people for advice if you were having a serious problem with depression?” and a list of options was provided. Next participants were asked: “If YOU were having difficulty with depression at some point in your life, how helpful would the following be?” and a list of service options was provided. Finally, participants were asked about past treatment experience.

#### Emotional Distress

Current emotional distress was assessed using the Kessler Psychological Distress Scale (K6), a validated measure of anxiety and depressive symptoms [31]. The 6-item survey asks: “During the past 30 days, about how often did you feel...nervous,...hopeless,...restless or fidgety,...so depressed that nothing could cheer you up,...that everything was an effort,...worthless.” Items were rated on a 5-point rating scale from 1 (none of the time) to 5 (all of the time). This measure has been found to be both valid and reliable, with a Cronbach alpha of .92 in previous research [31], .88 in the Clinic sample, and .91 in the Web sample.

### Statistical Methods

IBM SPSS statistics version 23.0 was used to conduct the data analysis. Demographic characteristics between the 2 samples were compared using independent samples *t* test for mean differences and chi-square comparisons for the proportions in the 2 groups. The information preferences questions’ 0-8 rating scales were categorized into three categories as follows: 0-2: not important, not likely, not preferred, not helpful; 3-5: moderately important, moderately likely, moderately preferred, moderately helpful; and 6-8: very important, very likely, very preferred, very helpful. The proportions of respondents providing high ratings (ie, very important, very likely, very preferred, or very helpful) on the information preferences questions as well as mean ratings with CIs are presented in Tables 2-5. CIs are often used in survey research and have been recommended rather than pairwise significance tests for comparisons between and within groups because they help the reader understand the magnitude of differences rather than simply concluding that a difference is statistically significant [32,33]. When making comparisons between means (ie, between groups and across different question items), it should be noted that in approximately one case out of 20, the 95% CIs will be nonoverlapping even in the absence of a difference in that measure within the underlying populations.

We conducted a linear regression analysis to explore the sociodemographic predictors of the number of information topics considered to be very important by participants in the Clinic sample with different characteristics. We were particularly interested in the relationships between information needs and previous experience with depression and its treatment as predictors.

## Results

### Participants

In the Clinic sample, the mean age of participants was 40 years, and it was reasonably well balanced for gender with 57.1% being female (136/238; Table 1). In terms of cultural background, 71.0% (169/238) of this sample were white, whereas 17.2% (41/238) were Aboriginal. The Web sample was similar to the Clinic sample in demographic characteristics; the mean age of participants was 39 years, 86.0% were white (241/280) and 12.1% (34/280) had an Aboriginal background. However, the Web sample had a much larger proportion of females (83.9%, 235/280). On average, respondents in both samples had completed 2 years of education after high school, and more than half had been working full-time in the prior year. Although Web surveys have the potential to reach a broader audience, almost all participants responding on the Web were from Manitoba (92.9%, 260/280). In the previous 12 months, 63.0% (150/238) of respondents in the Clinic sample indicated that they had searched the Internet for health-related information compared with 92.9% (260/280) in the Web group (χ^2^_1_=66.9, *P*<.001). Respondents rated how familiar they were with types of help available for depression on a 0-8 rating scale (not at all familiar to very familiar). More of those (55.0%, 154/280) in the Web sample indicated that they were very familiar with types of help for depression (rating of 6-8; mean 5.42, 95% CI 5.16-5.68), compared with 28.2% (67/238) in the Clinic sample (mean 3.70, 95% CI 3.38-4.02).

**Table 1:** Sample characteristics.

Characteristics		Clinic sample (N=238)	Web sample (N=280)	Statistical comparison	*P* value
Mean age (SD^a^)		39.0 (13.90)	40.2 (12.47)	*t*_508_=−0.98	.33	
**Gender proportion, n (%)**				χ^2^_1_=48.4	<.001^b^
	Female	136 (57.1)	235 (83.9)			
	Male	102 (42.9)	45 (16.1)		
**White, n (%)**				χ^2^_1_=17.6	<.001^b^
	Yes	169 (71.0)	241 (86.1)			
	No	69 (29.0)	39 (13.9)	
**Married, n (%)**				χ^2^_1_=5.1	.03^b^
	Yes	145 (60.9)	143 (51.1)	
	No	93 (39.1)	137 (48.9)	
Working full-time proportion, n (%)		143 (60.0)	148 (52.9)	χ^2^_1_=2.7	.10
Mean years education (SD)		13.8 (3.80)	14.1 (5.21)	*t*_516_=−0.58	.57
Distress score (SD)		5.1 (4.57)	10.5 (6.14)	*t*_463_=10.5	<.001^b^
Received counseling for depression, n (% yes)		83 (34.9)	224 (80.0)	χ^2^_1_=103.9	<.001^b^
Counseling for depression would have been helpful but not received, n (% yes)		88 (37.0)	210 (75.0)	χ^2^_1_=72.9	<.001^b^
Received medication for depression, n (% yes)		57 (24.0)	202 (72.1)	χ^2^_1_=112.6	<.001^b^
Medication for depression would have been helpful but not received, n (% yes)		33 (13.9)	106 (37.9)	χ^2^_1_=103.9	<.001^b^

^a^SD: standard deviation.

^b^Significantly different means or proportions between the 2 samples, where *P*<.05.

The Clinic sample was minimally distressed with an average K6 score of 5.1 (K6 sum scores range from 0 to 24). In contrast, the Web sample was significantly more distressed with a K6 score of 10.5 (*P*<.001). The recommended threshold for identifying a likely mental disorder is 13 or higher [34]. Approximately 80.0% (224/280) of the Web sample reported previously receiving counseling or therapy for depression at some time in their life, whereas 72.1% (202/280) reported receiving medication. This is compared with only 34.9% (83/238) and 24.0% (57/238) in the Clinic sample.

### Important Information Content When Considering Help

Table 2 shows ratings of importance of 20 information topics concerning the depression treatment. The overall impression is that both samples of respondents viewed most of the topics as very important. In both groups, participants placed a high level of importance on information about the effectiveness of treatment, goal, or outcome of treatment, how the treatment works, what happens when the treatment stops, and the advantages and disadvantages of a treatment approach. Mean ratings of importance as well as proportions rating a topic as very important were both greater overall in the Web sample compared with the Clinic sample. We also asked about seven other administrative topics (such as timing of appointments, hours of service, location of services). As these administrative issues will differ by geographic region, they are presented in Multimedia Appendix 3.

**Table 2:** Treatment options: What information would be important to you if you were considering help (for yourself, a close friend, or a close family member)?

Information type	Clinic sample (N=231)		Web sample (N=262)	
	Very important n (%)	Mean rating (95% CI)	Very important n (%)	Mean rating (95% CI)
All available treatments	164 (71.0)	6.4 (6.16-6.66)	233 (88.9)	7.2 (7.09-7.40)^a^
Available medication treatments	134 (58.0)	5.7 (5.47-6.03)	183 (69.9)	6.3 (6.11-6.57)^a^
Available counseling or psychological treatments	162 (70.1)	6.3 (6.06-6.58)	233 (88.9)	7.2 (7.06-7.40)^a^
Self-help treatment	88 (38.1)	4.8 (4.46-5.06)	204 (77.9)	6.7 (6.44-6.85)^a^
Herbal remedies	81 (35.1)	4.3 (4.00-4.63)	110 (42.0)	4.7 (4.37-4.95)
Exercise	157 (68.0)	6.0 (5.76-6.30)	194 (74.1)	6.5 (6.25-6.70)
Meditation	122 (52.8)	5.4 (5.07-5.65)	-^c^	-
Bright light therapy	104 (45.0)	4.9 (4.58-5.19)	173 (66.0)	5.8 (5.59-6.09)^a^
What you have to do as part of the treatment	164 (71.0)	6.3 (6.06-6.56)	228 (87.0)	7.1 (6.92-7.29)^a^
Cost of treatment to you	139 (60.2)	5.7 (5.38-6.01)	207 (79.0)	6.8 (6.55-7.01)^a^
Cost of treatment to health care system	81 (35.1)	4.3 (3.99-4.63)	68 (26.0)	3.8 (3.48-4.06)
Effectiveness of treatment	201 (87.0)	7.1 (6.85-7.25)	236 (90.1)	7.3 (7.12-7.45)
How treatment works	199 (85.6)	6.8 (6.60-7.05)	225 (85.9)	7.0 (6.85-7.21)
Goal or outcome of treatment	201 (86.1)	7.1 (6.86-7.26)	238 (90.8)	7.3 (7.12-7.43)
How long it takes for treatment to produce results	173 (74.9)	6.4 (6.11-6.60)	218 (83.2)	6.9 (6.69-7.07)^a^
How long treatment continues	168 (72.7)	6.3 (6.09-6.57)	215 (82.1)	6.8 (6.59-6.98)^a^
What happens when treatment stops	199 (86.1)	7.0 (6.74-7.16)	233 (88.9)	7.2 (6.99-7.34)
Common side effects of treatment	194 (84.0)	6.9 (6.70-7.10)	228 (87.0)	7.1 (6.95-7.30)
Uncommon but serious side effects of treatment	194 (84.0)	6.9 (6.67-7.10)	212 (80.9)	6.8 (6.62-7.01)
Advantages and disadvantages of treatment	189 (81.8)	6.8 (6.58-7.00)	233 (88.9)	7.1 (6.95-7.30)

^a^Web sample and Clinic sample CIs do not overlap.

^b^Each source was rated on a 9-point rating scale with the anchors 0-2 (not important), 3-5 (moderately important), and 6-8 (very important).

^c^“-” indicates items in Clinic but not Web survey.

### Preferred Source of Advice

In the Clinic sample, respondents reported that if they were having serious problems with depression, they would be very likely to speak with a romantic partner or spouse (63.2%, 146/231), a family doctor (60.2%, 139/231), or a counselor or therapist (58.9%, 136/231; see Table 3). Those in the Web sample showed similar preferences in general, although this group reported a higher likelihood of speaking to a counselor or therapist (80.2%, 210/262) as compared with a family doctor (69.9%, 183/262), or a romantic partner or spouse (61.1%, 160/262). Few people reported being very likely to speak with a religious leader or elder in either sample (15.2%, 35/231 and 17.2%, 45/262).

**Table 3:** How likely would you be to talk to one of the following people for advice if you were having a serious problem with depression?

Source of advice	Clinic sample (N=231)		Web sample (N=262)	
	Very likely n (%)	Mean rating (95% CI)	Very likely n (%)	Mean rating (95% CI)
Romantic partner or spouse	146 (63.2)	5.8 (5.53-6.14)	160 (61.2)	5.7 (5.33-5.97)
Parent	86 (37.2)	4.1 (3.76-4.52)	68 (26.0)	3.4 (3.10-3.78)
Family member (not parent)	74 (32.0)	4.0 (3.65-4.32)	76 (29.0)	3.8 (3.48-4.12)
Friend	106 (45.9)	5.0 (4.64-5.25)	139 (53.1)	5.2 (4.92-5.49)
Phone-in counseling or health line	69 (29.9)	3.8 (3.47-4.16)	81 (30.9)	3.99 (3.69-4.29)
Counselor or therapist	136 (58.9)	5.5 (5.21-5.82)	210 (80.2)	6.7 (6.43-6.88)^a^
Religious leader or community elder	35 (15.2)	2.1 (1.74-2.45)	45 (17.2)	2.1 (1.75-2.44)
Family doctor	139 (60.2)	5.7 (5.39-6.00)	183 (69.9)	6.1 (5.81-6.34)

^a^Web sample and Clinic sample CIs do not overlap.

^b^Each source was rated on a 9-point rating scale with the anchors 0-2 (not likely), 3-5 (moderately likely), and 6-8 (very likely).

### Preferred Method of Receiving Information

There are several ways to receive information about depression and its treatment. Table 4 shows that Clinic participants indicated a high level of preference for receiving information through discussion with a medical doctor (61.9%, 143/231), a counselor or therapist (61.0%, 141/231), or through a written information sheet or a website that could be accessed from home (50.2%, 116/231). The Web sample not only indicated a greater preference for a discussion with a counselor or therapist (74.1%, 194/262 very preferred) but also indicated preference for information in written form (61.8%, 162/262 very preferred) through discussion with a medical doctor (59.2%, 155/262) or a website accessed from home (56.9%, 149/262). Video information through a website had the lowest level of preference for both the Clinic and Web surveys (about 30% highly preferred).

**Table 4:** Preferred method of receiving information about services.

Preferred method	Clinic sample (N=231)		Web sample (N=262)	
	Very preferred n (%)	Mean rating (95% CI)	Very preferred n (%)	Mean rating (95% CI)
Written form (information sheet)	116 (50.2)	5.3 (5.02-5.59)	162 (61.8)	6.0 (5.76-6.27)^a^
Discussion with medical doctor	143 (61.9)	5.9 (5.62-6.10)	155 (59.2)	5.7 (5.43-5.91)
Discussion with counselor or therapist	141 (61.0)	5.7 (5.44-6.00)	194 (74.1)	6.2 (6.01-6.46)^a^
Video on the Web	69 (29.9)	4.0 (3.67-4.32)	81 (30.9)	4.2 (3.93-4.49)
Recommended website accessed from home	116 (50.2)	5.0 (4.70-5.35)	149 (56.9)	5.6 (5.37-5.85)^a^

^a^Web sample and Clinic sample CIs do not overlap.

^b^Each source was rated on a 9-point rating scale with the anchors 0-2 (not preferred), 3-5 (moderately preferred), and 6-8 (very preferred).

### Helpfulness of Various Forms of Assistance

In considering various forms of assistance for depression, many approaches to treatment were seen as likely to be very helpful by clinic respondents including in-person meetings with a counselor (68.8%, 159/231), exercise (66.2%, 153/231), and medication recommended by a psychiatrist (51.0%, 118/231; Table 5). A similar pattern of responses was found for the Web sample, but that group provided higher ratings of the helpfulness for most forms of assistance. (Note that the Web survey did not ask about exercise, meditation, herbal medication, or bright light therapy.)

**Table 5:** How helpful would the following types of assistance be if you were having a problem with depression?

Type of assistance	Clinic sample (N=231)		Web sample (N=262)	
	Very helpful n (%)	Mean rating (95% CI)	Very helpful n (%)	Mean rating (95% CI)
Recommended self-help book	72 (31.2)	4.3 (3.98-4.57)	113 (43.1)	4.9 (4.65-5.20)^a^
Recommended self-help website	81 (35.1)	4.5 (4.17-4.76)	109 (41.6)	5.1 (4.87-5.39)^a^
Telephone meetings with a counselor	86 (37.2)	4.4 (4.07-4.67)	131 (50.0)	5.0 (4.72-5.29)^a^
In person meetings with a counselor	159 (68.8)	6.1 (5.81-6.36)	223 (85.1)	6.9 (6.72-7.13)^a^
Educational meeting (about 2 h with 20-30 people)	65 (28.1)	4.0 (3.62-4.29)	94 (40.5)	4.2 (3.90-4.54)
Educational workshop (about 6 h with 20-30 people)	55 (23.8)	3.6 (3.25-3.90)	102 (38.9)	4.1 (3.81-4.47)
Web-based discussion group led by professional	42 (18.2)	3.2 (2.85-3.47)	63 (24.1)	3.7 (3.42-3.99)
Web-based discussion group led by person who has coped with depression	55 (23.8)	3.5 (3.18-3.82)	79 (30.2)	3.8 (3.51-4.11)
Medication recommended by your family doctor	109 (47.2)	4.9 (4.54-5.15)	147 (56.1)	5.4 (5.15-5.71)^a,b^
Medication recommended by a specialist in psychiatry	118 (51.1)	5.0 (4.72-5.34)	170 (64.9)	5.9 (5.57-6.14)^a^
Taking herbal medication	72 (31.2)	3.9 (3.55-4.21)	-^c^	-
Doing exercise	153 (66.2)	6.0 (5.69-6.22)	-	-
Doing meditation	109 (47.2)	4.9 (4.55-5.19)	-	-
Having bright light therapy	62 (26.8)	3.7 (3.37-4.04)	-	-

^a^Web sample and Clinic sample CIs do not overlap.

^b^Upon examination of the CIs with 3 decimal places, the CIs of the two samples do not overlap.

^c^“-” indicated items were in Clinic but not Web survey.

^d^Each source was rated on a 9-point rating scale with the anchors 0-2 (*not helpful*), 3-5 (*moderately helpful*), and 6-8 (*very helpful*).

### Web Respondents Who Did and Did Not Receive an Honorarium

We evaluated the impact of the introduction of an honorarium to increase recruitment for the Web sample by comparing the subsamples before and after the introduction of the honorarium. The samples that received and did not receive an honorarium were very similar in demographic characteristics (see Multimedia Appendix 4). One noteworthy difference was that a higher proportion of males responded after the introduction of the honorarium, although there continued to be a high proportion of female respondents. Similar mean ratings and pattern of responses were also found for the information needs and preferences questions for both those who received the honorarium and those who did not receive it (see Multimedia Appendices 5-8). By adding the honorarium we were able to double our participation in half the time (1 month), which is consistent with previous research on improving response to Web- and paper-based surveys [35,36].

### Predictors of Information Topics Considered Very Important

Table 6 describes the regression analysis for predictors of the number of information topics considered to be very important by participants in the Clinic sample. We focused on the Clinic sample because it was more diverse in terms of gender and ethnic background. The partial correlation (*pr*) reported in the table, when squared, indicates the unique proportion of the variance in the outcome that is accounted for by each predictor variable when all other predictors and their shared variance have been accounted for in the model. Gender (beta=−1.94, *P*=.007, *pr*=−.19), ethnicity (beta=1.85, *P*=.02, *pr*=.17), therapy received or needed (beta=2.07, *P*=.03, *pr*=.16), and medication received or needed (beta=−2.78, *P*=.005, *pr*=−.20) were found to be significant predictors of number of information topics after accounting for marital status, age, education, and distress level. Overall the females indicated more information topics as important than males (13.8 information topics as very important vs 12.2), the white respondents saw more topics as important than those from other groups (13.6 vs 11.8), those who had received or needed therapy saw more topics as important than those who had not (13.6 vs 12.8), and those who received or needed medication saw fewer topics as important than those who had not (12.6 vs 13.4). The reader should note that the magnitude of the difference in amount of information desired by the different demographic groups is small and that personal preferences may play a stronger role here than demographic characteristics [3].

**Table 6:** Predictors of composite information topic score for topics given a very important rating for the Clinic sample.

Predictor	*B* ^a^	SE *B*^b^	Beta^c^	*P* value	*pr* ^d^
Gender (0=female, 1=male)	−1.94	.72	−.19	.007	−.19
Ethnicity (0=nonwhite, 1=white)	1.85	.76	.17	.02	.17
Marital status (0=not married, 1=married)	.47	.76	.05	.54	.04
Age	.004	.03	.01	.88	.01
Education sum	−.02	.09	−.02	.83	−.02
Distress score	−.11	.09	−.10	.21	−.09
Therapy received or needed^e^	2.07	.92	.21	.03	.16
Medication received or needed	−2.78	.98	−.25	.005	−.20

^a^*B*: unstandardized coefficients (weights).

^b^SE *B*: standard error of unstandardized coefficient.

^c^Beta: standardized coefficients (weights).

^d^*pr*: partial correlation.

^e^Therapy received or needed includes individuals who indicated that they had previously received counseling or therapy for depression in the past or there was a time that they would have benefited from counseling or therapy but did not receive it.

^f^Medication received or needed includes individuals who indicated that they had previously received medication for depression in the past or there was a time that they would have benefited from medication but did not receive it.

^g^This includes the Clinic sample (N=231) data only. Information importance composite score was calculated by summing the topics that respondents provided a rating of 6-8 (very important). The range of scores on this variable is from 0 to 20.

## Discussion

### Principal Findings

In considering how typical respondents in the Clinic and Web surveys were of people living in the region, we compared characteristics of survey respondents with people living in the city of Winnipeg (population of about 700,000) and to those living in the province (population of about 1.3 million). Most of the Clinic participants would live in Winnipeg, whereas persons visiting the websites could have come from anywhere in the province. The Clinic sample is primarily from white (71.0%) and Aboriginal or First Nations (16.5%) cultural groups. Manitoba has an Aboriginal population of 14% [37], whereas Winnipeg has an Aboriginal population of 11% [38]. The Clinic sample, which was much more balanced for gender compared with the Web sample, had slightly more females than the general population of Manitoba and Winnipeg, which are 50% and 51% female, respectively [39,40]. There were smaller proportions of individuals in both samples who were working full-time compared with the general population (79% in the Manitoba population are working full-time; [41]). Both samples were similarly educated compared with the population of Manitoba with an average of 2 years of postsecondary education. It was found that 88% of the Manitoba population (aged 25-64 years) has attained a high school diploma or equivalent [42]. There were slightly more individuals in the two samples that indicated that they were married or living together in a marital like relationship (common law) compared with a rate of 46% in the general population of Manitoba and Winnipeg [40,43].

The Clinic sample reported less current distress and had less experience with previous treatments for depression than the Web sample. This is understandable because the Clinic sample was recruited from people seeking general medical assessment and treatment, whereas those visiting the self-help association websites were more likely focused on getting information on depression and anxiety. Furthermore, persons who have sought help in the past are more likely to seek help in the future [44]. In considering the higher proportion of females in the Web sample than the Clinic sample, possible explanations may be the higher prevalence of depression among females [45] and the greater tendency of them to seek help [46]. In the Clinic sample, we also found that females judge information on more topics to be very important.

The Clinic sample appears to produce more cultural diversity that is a closer match to the population. Both surveys had an underrepresentation of males relative to the population. In the case of the Web survey, this was improved somewhat by the use of an honorarium to encourage participation.

We found that in both Clinic and Web samples, people are interested in information on a wide range of topics. Participants were especially interested in psychological treatments, physical exercise, and medication treatments. Characteristics of treatments such as the effectiveness of treatments, their goals, duration, side effects, and what happens when treatment stops were also considered to be important. This finding that people are interested in information on many topics is consistent with previous research on mental health information needs and preferences [47,48].

One can imagine how difficult it would be to review this amount of information in the typical primary care visit of 10-15 min and even in a specialist visit of 20-50 min. More importantly from the patient’s perspective it would also be very challenging to remember this amount of information if it were presented orally, especially when struggling with depression. In these situations, it is often helpful for the clinician to provide information in some form that can be reviewed over a longer time period by the patient and concerned family and friends. This type of written information is commonly provided in the form of patient-oriented brochures [13] or Web-based information [14]. Even in text format, it would take considerable space to address all of the topics identified as important and to put this in the context of the quality of the scientific evidence available. One way of dealing with differences in preference among individuals for more or less information is to produce information focused on each topic and allow information users to choose the areas of information that are of most interest to them.

Other researchers [47] have found that Web-based resources about depression are reasonably good, although these researchers did not present information on the specific content areas covered by these websites. Current resources tend to describe the diagnosis and some of the treatments available but they provide little or no evidence-based information to answer most of the questions identified as important in this survey. The shortcoming in Web-based information is not limited to information concerning depression, but is also seen in information concerning other mental health problems such as children’s anxiety [12], and medical conditions such as inflammatory bowel disease [11]. A challenge for those developing information resources is that there is a limited amount of evidence available to answer some of these questions and some of the information is difficult for professionals to access. Whereas there is a wide range of evidence concerning the effectiveness of psychological and pharmacological treatments for depression, there is little research available on self-help approaches, herbal remedies, exercise, meditation, and bright light therapy. Members of the public would have difficulty locating and evaluating the quality of this evidence. It would be valuable to take a knowledge synthesis approach [48] to review the evidence available to answer these questions and to provide information in a form that would be clear for the public and for health professionals.

A specific example of challenges in accessing evidence to answer an important question is the topic of what happens when psychological or medication treatment stops. Many medication treatment trials are of relatively short duration (eg, 8-12 weeks), include no follow-up period, and report no data on what happens after medication is discontinued. Psychological treatment trials often report follow-up after treatment is terminated but the time period is often limited (6-12 months; [49]). Studies including longer follow-up after treatment is discontinued, suggest that return of symptoms after treatment is discontinued is a common experience [50]. These studies would be difficult for the layperson and even a reasonably well-informed professional to locate and digest. Again, a knowledge synthesis approach of reviewing information, assessing the quality of research, and summarizing the information in clear language would be very helpful.

Ratings of importance of most topics were both greater overall in the Web sample compared with the Clinic sample. This is not surprising as those in the Web survey were seeking information, whereas those attending the clinic would have been seeking care for a wide range of health problems. The Web sample also reported higher levels of psychological distress, which could be associated with a higher interest in depression information.

In considering people to speak to for advice, respondents reported a broad range of people that were seen as important sources of advice. Counselors and family doctors were seen as important sources of advice along with romantic partners and friends. In the Web group, a counselor or therapist was rated particularly highly as a source of advice. This may have been related to the high amount of experience in this group with counseling for depression.

Participants in both samples indicated preferences for receiving information in a variety of ways including discussion with a counselor or therapist, written form (such as a brochure), and discussion with a medical doctor. Despite being Web users, receiving information in written form or brochure was highly rated in the respondents to the Web survey. These findings demonstrate the importance of having information available to be delivered via different formats or methods, which is consistent with previous research in this area [3,51]. People do not have to choose a single source of information, and brochure or Web-based information can complement discussion with a health service provider and vice versa.

Overall, the pattern of responses on the helpfulness of assistance types between the Clinic and Web samples was quite similar. However, the Web sample provided higher ratings of helpfulness of most assistance types. The Web sample was more distressed and had more treatment experience so they may have seen treatment options as more helpful for this reason. In both samples, counseling or therapy was rated highest among the different forms of assistance, which is not surprising given the literature on preference for psychological treatments [52]. Medication as a treatment for depression has also been widely studied, has been shown to be effective, and is widely available [53]. Therefore, it is reasonable that many respondents also provided high ratings for medication recommended by a family doctor or psychiatrist. Self-help approaches to treating depression were rated significantly higher by the Web sample compared with the Clinic sample. As the Web sample participated in this survey by accessing self-help association websites, it is not surprising that they would be interested in self-help methods of treatment. Self-help resources are advantageous in that they are potentially widely available and usually associated with lower cost [54].

When we considered characteristics of respondents related to the number of information topics considered to be very important, we found that females, whites, and those who had received or felt they would have benefited from therapy in the past saw more topics as very important. Those who had received or thought that they would have benefited in the past from medication treatment saw fewer topics as important. The magnitude of these differences was modest however. This finding was similar to findings by Cunningham and colleagues [3] in a large survey with more than 1000 respondents from primary care clinics. Cunningham [3] found that there were larger differences based on patterns of information preferences and suggested that the best solution is to make information available in a variety of formats (paper and Web formats) in a variety of settings, allowing people to choose the type of information they prefer. Taken together, the results suggest that both persons coping with depression and persons seeking information about depression would be interested in information developed to answer important questions concerning depression treatment. It is likely that information needs for other common mental health problems would be quite similar but this should be the subject of future research. Guidelines about the development and evaluation of health information for the public are available from the International Patient Decision Aids Standards collaboration [55]. The wide range of information topics judged to be important by members of the public suggests that it would be very difficult to address these information needs via oral communication during health care visits or using currently available materials. A resource with Web-based information and downloadable fact sheets has the advantage that it can provide information in a format that can be accessed by the public (searching for information for themselves or family members) and by health professionals interested in information to use to supplement discussions with their patients. Our team has been developing resources to address these needs with material address each of the main topics identified as important by community members. This resource focused on information for Canadians is available on the Web [56]. This information has been evaluated favorably by service providers in primary care settings [4]. Much of this information would be suitable for the public in many countries. National and regional information would be particularly helpful around questions concerning cost of treatment and resources available to support consumers in paying for treatment. The topics concerning the administrative aspects of treatment (health care providers providing treatment, waiting periods, location of services, hours) were also considered to be very important by many respondents. It is necessary to tailor this information at the regional and local level.

### Limitations

This study has a few main limitations. One major limitation is the differences in the characteristics of the samples. Therefore, some of the differences found in the results may have been a consequence of the different make-up of the two samples. Recruiting more similar samples would have allowed for more control of potential sample effects. A second limitation is that the response rate for the Web survey is unknown. Due to a link to the survey being available on a number of websites, we do not know who might have reviewed the invitation to participate in the survey, and not clicked on the link to start completing the survey. In comparison, 71% of the people approached for the paper-based survey agreed to participate. Another limitation is that most of the respondents to the Web-based survey were female (84%). This limits the generalizability of those findings. However, we compared the results reported by males and females within the Clinic survey (57% female) and the response patterns were very similar (data not shown). The final limitation is related to the Clinic survey. Participants were recruited from primary health care settings and their opinions may not be generalizable to the opinions of the general public.

### Conclusions

This is one of few studies that addresses the information needs and preferences concerning treatment options for depression. The findings may help practitioners in making resources available that assist members of the public in decision making. Each survey format has its advantages. The Clinic survey includes a more broad and representative sample. The Web survey through self-help association websites captures individuals who are clearly seeking information. Web surveys are considerably lower in cost than a survey administered by a research assistant inviting participation by visitors to a primary care medical clinic. The use of an honorarium to encourage participation increases response rate and likely representativeness of the sample (compared with the population at large), although it also increases the cost. The similarities in the broad findings between the Clinic and the Web surveys is reassuring and suggests that helpful opinions may be gathered by each method as long as the limitations of the sampling approach are recognized.

## Appendix

Multimedia Appendix 1 Website survey notice.

Multimedia Appendix 2 Complete version of survey.

Multimedia Appendix 3 Administrative aspects of treatment.

Multimedia Appendix 4 Sociodemographic characteristics of respondents who received or did not receive honorarium.

Multimedia Appendix 5 Treatment options: what information would be important to you if you were considering help? Responses with or without honorarium.

Multimedia Appendix 6 How likely would you be to talk to one of the following people for advice if you were having a serious problem with depression? Responses with and without honorarium.

Multimedia Appendix 7 Preferred method of receiving information about services. Responses with and without honorarium.

Multimedia Appendix 8 How helpful would the following types of assistance be if you were having a problem with depression? Responses with and without honorarium.

## Abbreviations

DSM: Diagnostic and Statistical Manual of Mental Disorders
REB: Research Ethics Board
SD: standard deviation
