# Supplementary material for: Gathering Opinions on Depression Information Needs and Preferences: Samples and Opinions in Clinic Versus Web-Based Surveys
Source: JMIR Ment Health. 2017 Apr 24;4(2):e13. doi: 10.2196/mental.7231 (PMC5422653; doi:10.2196/mental.7231)
Supplement: Multimedia Appendix 1 [file mental_v4i2e13_app1.pdf]

Multimedia Appendix 1

Survey Recruitment Notice

Survey: People often have difficulty finding answers to their questions about treatment options for depression.

A research team at the University of Manitoba is conducting a survey about people's information needs and preferences.

The survey is anonymous.

People who complete the survey will be able to download fact sheets, which answer common questions about treatment options for depression. They will also receive a gift card for $10 for a choice of coffee shops (Tim Horton's, Starbucks) or grocery stores (Safeway, Superstore/Loblaws, Sobeys).

If you provide your mailing address to receive the gift card, this information will not be associated with your responses. Your name and address will not be used for any other reason and will be deleted 3 months after the gift card has been mailed.

Your opinions are important. The more people who respond to the survey – the greater our understanding of the information needs of different people.

If you wish to complete the survey please click the button below to be taken to the website for the survey.

[GO TO THE SURVEY]
