# Supplementary material for: Gathering Opinions on Depression Information Needs and Preferences: Samples and Opinions in Clinic Versus Web-Based Surveys
Source: JMIR Ment Health. 2017 Apr 24;4(2):e13. doi: 10.2196/mental.7231 (PMC5422653; doi:10.2196/mental.7231)
Supplement: Multimedia Appendix 2 [file mental_v4i2e13_app2.pdf]

Multimedia Appendix 2

Complete Version of Survey

Information Preferences Questionnaire

Background Information: Please mark your answer or fill in the bubble.

1. Today's Date: Day_____ Month__________ Year 2014

2. Your Age: ________

3. Gender: Male O (1) **or Female O** (2)

4. Which of the following best describes your marital status?
O currently married or living with someone in marital-like relationship (1)
O never married & never lived with someone in a marital-like relationship (2)
O separated (3)
O divorced or formerly lived with someone in a marital-like relationship (4)
O widowed (5)

5. In the past 12 months, what were you doing most of the time?
O Working (full time) (1) O Retired (6)
O Working (part time) (2) O Disabled (7)
O Homemaking (3) O Something else (8). Please describe: __________
O School (4) ________________________________
O Work half time/school half time (5) ________________________________

6. Education: How many years of education have you completed in the following areas?
Grade School: O0 O1 O2 O3 O4 O5 O6 O7 O8 O9 O10 O11 O12 O13
College, technical, business, vocational, nursing, apprenticeship program (non-university): O0 O1 O2 O3 O4 O5 O6
University program: O0 O1 O2 O3 O4 O5 O6 O7 O8 O9 O10 O11 O12

9. People living in Canada come from many different cultural and racial backgrounds (Please mark all that apply to your background).
O White or Caucasian (1) O Black (2) O Chinese (3)
O Japanese (4) O Korean (5)
O Aboriginal (North American Indian, Métis or Inuit) (6)
O Southeast Asian (e.g., Cambodian, Indonesian, Laotian, Vietnamese) (7)
O South Asian (e.g., East Indian, Pakistani, Sri Lankan) (8)
O Middle Eastern (e.g., Afghan, Iranian) (9)
O Filipino (10)
O Latin American (11)

O Arab (12)
O Other (Please Specify:__________________) (13)

The following questions ask about how you have been feeling during the **past 30 days.** For each question, please mark the rating that best describes how often you had this feeling.

During the past 30 days, about how often did you feel …	All of the time (1)	Most of the time (2)	Some of the time (3)	A little of the time (4)	None of the time (5)
1. …nervous?	O	O	O	O	O
2. …hopeless?	O	O	O	O	O
3. …restless or fidgety?	O	O	O	O	O
4. …so depressed that nothing could cheer you up?	O	O	O	O	O
5. …that everything was an effort?	O	O	O	O	O
6. …worthless?	O	O	O	O	O

Many adults have difficult problems with depression at times. In fact, over any year about 1 in 10 people are having problems with depression. At times people decide they would like to look for assistance beyond the usual help that can be provided by their friends and family.

Here is the story of a person who is having problems with depression.

Steven is a man who has been feeling unusually sad and miserable for the last few weeks. He is tired all the time and has trouble sleeping at night. He doesn't feel like eating and has lost weight. He can't keep his mind on his work and his boss has not been too pleased with him. He puts off making any decisions and even day-to-day tasks seem too much for him. His parents and co-workers are very concerned about him.

Information Preferences

1. a. DURING THE PAST 12 MONTHS, have you used the Internet to search for medical or health-related information?

O Yes O No

b. If yes, how often do you use the Internet to search for medical or health-related information? Please circle the number that best fits.

O	1	2	3	4	5
Prefer not to respond	Not at all	A little	Moderately	Quite a lot	A great deal

2. Please circle the number that best fits your rating from 0 to 8.

How familiar are you with the types of help available for depression?

O 0--------1--------2--------3--------4--------5--------6--------7--------8

Prefer not to respond	Not at all familiar	Moderately familiar	Very familiar

At some time in your life you, a close friend, or a close family member might be having a problem with depression. What information would be important to you in considering the kinds of help available for depression? (Please circle the number that best fits).

3. All of the types of treatment available for depression.

O 0--------1--------2--------3--------4--------5--------6--------7--------8

Prefer not to respond	Not at all important	Moderately important	Very important

4. The various medication treatments for depression.

O 0--------1--------2--------3--------4--------5--------6--------7--------8

Prefer not to respond	Not at all important	Moderately important	Very important

5. The various counseling or psychological treatments for depression.

O 0--------1--------2--------3--------4--------5--------6--------7--------8

Prefer not to respond	Not at all important	Moderately important	Very important

6. Self-help treatment for depression (self-help book or website).

O	0--------1--------2--------3--------4--------5--------6--------7--------8		
Prefer not to respond	Not at all important	Moderately important	Very important

7. Herbal remedies for depression (such as St. John's wort, or something from a health foods store).

O	0--------1--------2--------3--------4--------5--------6--------7--------8		
Prefer not to respond	Not at all important	Moderately important	Very important

8. Exercise as a treatment for depression.

O	0--------1--------2--------3--------4--------5--------6--------7--------8		
Prefer not to respond	Not at all important	Moderately important	Very important

9. Meditation as a treatment for depression.

O	0--------1--------2--------3--------4--------5--------6--------7--------8		
Prefer not to respond	Not at all important	Moderately important	Very important

10. Bright light therapy for seasonal depression (seasonal affective disorder).

O	0--------1--------2--------3--------4--------5--------6--------7--------8		
Prefer not to respond	Not at all important	Moderately important	Very important

11. What you have to do as part of the treatment.

O	0--------1--------2--------3--------4--------5--------6--------7--------8		
Prefer not to respond	Not at all important	Moderately important	Very important

12. The cost of the treatment to you (if any).

O	0--------1--------2--------3--------4--------5--------6--------7--------8		
Prefer not to respond	Not at all important	Moderately important	Very important

13. The cost of the treatment to the health care system.

O 0--------1--------2--------3--------4--------5--------6--------7--------8
Prefer not Not at all Moderately Very
to respond important important important

14. The effectiveness or success of the treatment.

O 0--------1--------2--------3--------4--------5--------6--------7--------8
Prefer not Not at all Moderately Very
to respond important important important

15. How the treatment works.

O 0--------1--------2--------3--------4--------5--------6--------7--------8
Prefer not Not at all Moderately Very
to respond important important important

16. The goal or outcome of treatment.

O 0--------1--------2--------3--------4--------5--------6--------7--------8
Prefer not Not at all Moderately Very
to respond important important important

17. How long it takes for the treatment to produce results.

O 0--------1--------2--------3--------4--------5--------6--------7--------8
Prefer not Not at all Moderately Very
to respond important important important

18. How long the treatment continues.

O 0--------1--------2--------3--------4--------5--------6--------7--------8
Prefer not Not at all Moderately Very
to respond important important important

19. What happens when the treatment stops.

O 0--------1--------2--------3--------4--------5--------6--------7--------8
Prefer not Not at all Moderately Very
to respond important important important

20. The common side effects of the treatment.

O	0--------1--------2--------3--------4--------5--------6--------7--------8		
Prefer not to respond	Not at all important	Moderately important	Very important

21. The uncommon, but serious side effects of the treatment.

O	0--------1--------2--------3--------4--------5--------6--------7--------8		
Prefer not to respond	Not at all important	Moderately important	Very important

22. The advantages (pros) and disadvantages (cons) of each type of treatment.

O	0--------1--------2--------3--------4--------5--------6--------7--------8		
Prefer not to respond	Not at all important	Moderately important	Very important

23. The training and profession of the person providing treatment (e.g., family doctor, nurse, occupational therapist, psychiatrist, psychologist, social worker, counselor)?

O	0--------1--------2--------3--------4--------5--------6--------7--------8		
Prefer not to respond	Not at all important	Moderately important	Very important

24. The health-care provider's experience in treating these problems.

O	0--------1--------2--------3--------4--------5--------6--------7--------8		
Prefer not to respond	Not at all important	Moderately important	Very important

25. The waiting period before starting the treatment.

O	0--------1--------2--------3--------4--------5--------6--------7--------8		
Prefer not to respond	Not at all important	Moderately important	Very important

26. Where treatment will take place.

O	0--------1--------2--------3--------4--------5--------6--------7--------8		
Prefer not to respond	Not at all important	Moderately important	Very important

27. The amount of your time that is required to take the treatment (for appointments and so on).

O	0--------1--------2--------3--------4--------5--------6--------7--------8		
Prefer not to respond	Not at all important	Moderately important	Very important

28. The time of day that the appointments would be scheduled (e.g., morning, afternoon, after school or work, evening).

O	0--------1--------2--------3--------4--------5--------6--------7--------8		
Prefer not to respond	Not at all important	Moderately important	Very important

29. The treatment options that a health-care provider recommends for this situation and the reasons why.

O	0--------1--------2--------3--------4--------5--------6--------7--------8		
Prefer not to respond	Not at all important	Moderately important	Very important

30. Is there any other type of information about treatments for depression that you think is important?

____
____
____
___

Amount of Information

<u>Instructions</u>**: In making decisions about services, many people find it helpful to have information about the choices available. This information may be provided in different ways (such as brochures, discussion with a service provider, Internet) and in different amounts of detail.**
If you were considering help for yourself, a close friend, or a family member for problems with depression:
i) Please indicate (by circling) the amount of information you would prefer regarding these treatments.

1. Information about medication treatment.

O 0------------1-------------2------------3-------------4------------5

Prefer not To respond | 0 pages (none) | 2 pages | 4 pages | 6 pages | 8 pages | 10 pages or more

2. Information about counseling or psychological treatments.

O 0-------------1-------------2-------------3-------------4-------------5
Prefer not To respond | 0 pages (none) | 2 pages | 4 pages | 6 pages | 8 pages | 10 pages or more

3. Information about self-help approaches – such as self-help books or Internet based self-help programs.

O 0-------------1-------------2-------------3-------------4-------------5
Prefer not To respond | 0 pages (none) | 2 pages | 4 pages | 6 pages | 8 pages | 10 pages or more

4. Information about herbal remedies (such as St. John's wort).

O 0-------------1-------------2-------------3-------------4-------------5
Prefer not To respond | 0 pages (none) | 2 pages | 4 pages | 6 pages | 8 pages | 10 pages or more

5. Information about exercise as treatment for depression.

O 0-------------1-------------2-------------3-------------4-------------5
Prefer not To respond | 0 pages (none) | 2 pages | 4 pages | 6 pages | 8 pages | 10 pages or more

6. Information about bright light therapy for seasonal depression.

O 0-------------1-------------2-------------3-------------4-------------5
Prefer not To respond | 0 pages (none) | 2 pages | 4 pages | 6 pages | 8 pages | 10 pages or more

7. Information about meditation as treatment for depression.

O 0-------------1-------------2-------------3-------------4-------------5
Prefer not To respond | 0 pages (none) | 2 pages | 4 pages | 6 pages | 8 pages | 10 pages or more

Source of Information

Please indicate by circling how likely you would be to talk to one of the following people for advice if you were having a serious problem with depression?

1. A romantic partner/spouse?

O 0--------1--------2--------3--------4--------5--------6--------7--------8
Prefer not to respond | Not at all likely | Moderately likely | Very likely

2. One of your parents?

O 0--------1--------2--------3--------4--------5--------6--------7--------8
Prefer not to respond | Not at all likely | Moderately likely | Very likely

3. A family member (who is not a parent)?

O 0--------1--------2--------3--------4--------5--------6--------7--------8
Prefer not to respond | Not at all likely | Moderately likely | Very likely

4. A friend?

O 0--------1--------2--------3--------4--------5--------6--------7--------8
Prefer not to respond | Not at all likely | Moderately likely | Very likely

5. Someone on a phone-in counseling or health line?

O 0--------1--------2--------3--------4--------5--------6--------7--------8
Prefer not to respond | Not at all likely | Moderately likely | Very likely

6. A counselor or therapist?

O 0--------1--------2--------3--------4--------5--------6--------7--------8
Prefer not to respond | Not at all likely | Moderately likely | Very likely

7. Your religious leader or community elder?

O 0--------1--------2--------3--------4--------5--------6--------7--------8
Prefer not to respond | Not at all likely | Moderately likely | Very likely

8. Your family doctor?

O 0--------1--------2--------3--------4--------5--------6--------7--------8
Prefer not to respond | Not at all likely | Moderately likely | Very likely

Format of Information

Information about services can be provided in a number of ways. Please indicate (by circling) how you would prefer to receive information about various services.

1. Information given in written form (in an information sheet or booklet which you could take with you).

O	0--------1--------2--------3--------4--------5--------6--------7--------8		
Prefer not to respond	Not at all preferred	Moderately preferred	Very much preferred

2. Information given through discussion with a medical doctor.

O	0--------1--------2--------3--------4--------5--------6--------7--------8		
Prefer not to respond	Not at all preferred	Moderately preferred	Very much preferred

3. Information given through discussion with a counselor or therapist.

O	0--------1--------2--------3--------4--------5--------6--------7--------8		
Prefer not to respond	Not at all preferred	Moderately preferred	Very much preferred

4. Information provided by a video on the Internet.

O	0--------1--------2--------3--------4--------5--------6--------7--------8		
Prefer not to respond	Not at all preferred	Moderately preferred	Very much preferred

5. Information on a recommended Internet website which could be accessed and printed from home.

O	0--------1--------2--------3--------4--------5--------6--------7--------8		
Prefer not to respond	Not at all preferred	Moderately preferred	Very much preferred

6. Is there any other source of information that you would find helpful?

____
____
____
___

If YOU were having difficulty with depression at some point in your life, how helpful would the following be?

1. A recommended self-help book providing education and coping strategies to deal with depression.

O	0--------1--------2--------3--------4--------5--------6--------7--------8		
Prefer not to respond	Not at all helpful	Moderately helpful	Very helpful

2. A recommended self-help website providing education and coping strategies to deal with depression.

O	0--------1--------2--------3--------4--------5--------6--------7--------8		
Prefer not to respond	Not at all helpful	Moderately helpful	Very helpful

3. Scheduled telephone conversation with a counselor to discuss your depression and work on coping strategies.

O	0--------1--------2--------3--------4--------5--------6--------7--------8		
Prefer not to respond	Not at all helpful	Moderately helpful	Very helpful

4. In person meetings with a counselor/psychologist/therapist to discuss your depression and work on coping strategies.

O	0--------1--------2--------3--------4--------5--------6--------7--------8		
Prefer not to respond	Not at all helpful	Moderately helpful	Very helpful

5. Attending an educational meeting or seminar (about two hours with 20 to 30) people providing education and coping strategies to deal with depression.

O	0--------1--------2--------3--------4--------5--------6--------7--------8		
Prefer not to respond	Not at all helpful	Moderately helpful	Very helpful

6. Attending an educational workshop (about six hours with about 20 to 30) people providing education and coping strategies to deal with depression.

O 0--------1--------2--------3--------4--------5--------6--------7--------8
Prefer not to respond | Not at all helpful | Moderately helpful | Very helpful

7. Visiting an Internet discussion group led by a professional about coping with depression.

O 0--------1--------2--------3--------4--------5--------6--------7--------8
Prefer not to respond | Not at all helpful | Moderately helpful | Very helpful

8. Visiting an Internet discussion group led by a person who has coped with depression him or herself.

O 0--------1--------2--------3--------4--------5--------6--------7--------8
Prefer not to respond | Not at all helpful | Moderately helpful | Very helpful

9. Taking a medication to help with depression recommended by your family doctor.

O 0--------1--------2--------3--------4--------5--------6--------7--------8
Prefer not to respond | Not at all helpful | Moderately helpful | Very helpful

10. Taking a medication to help with depression recommended by a specialist in psychiatry.

O 0--------1--------2--------3--------4--------5--------6--------7--------8
Prefer not to respond | Not at all helpful | Moderately helpful | Very helpful

11. Taking a herbal medication (like St. John's wort) to help with depression.

O 0--------1--------2--------3--------4--------5--------6--------7--------8
Prefer not to respond | Not at all helpful | Moderately helpful | Very helpful

12. Doing regular exercise to help with depression.

O 0--------1--------2--------3--------4--------5--------6--------7--------8
Prefer not to respond | Not at all helpful | Moderately helpful | Very helpful

13. Doing regular meditation to help with depression.

O 0--------1--------2--------3--------4--------5--------6--------7--------8

Prefer not to respond | Not at all helpful | Moderately helpful | Very helpful

14. Using daily bright light therapy to help with depression.

O 0--------1--------2--------3--------4--------5--------6--------7--------8

Prefer not to respond | Not at all helpful | Moderately helpful | Very helpful

15. Thinking about your past experiences, was there a time when you received counseling or therapy from a professional (such as a counselor, therapist, or doctor) for depression?

O Yes (1) O No (2) O Not sure (3) O Prefer not to respond (4)

16. Thinking about your past experiences, was there a time when it would have been helpful to receive counseling or therapy from a professional (such as a counselor, therapist, or doctor) for depression, but you did not receive it?

O Yes (1) O No (2) O Not sure (3) O Prefer not to respond (4)

17. Thinking about your past experiences, was there a time when you received medication prescribed by a doctor for depression?

O Yes (1) O No (2) O Not sure (3) O Prefer not to respond (4)

18. Thinking about your past experiences, was there a time when it would have been helpful to take medication prescribed by a doctor for depression, but you did not receive it?

O Yes (1) O No (2) O Not sure (3) O Prefer not to respond (4)

Thank you very much for completing this questionnaire!!
