# Supplementary material for: Gathering Opinions on Depression Information Needs and Preferences: Samples and Opinions in Clinic Versus Web-Based Surveys
Source: JMIR Ment Health. 2017 Apr 24;4(2):e13. doi: 10.2196/mental.7231 (PMC5422653; doi:10.2196/mental.7231)
Supplement: Multimedia Appendix 3 [file mental_v4i2e13_app3.pdf]

Multimedia Appendix 3

Administrative Aspects of Treatment: What information would be important to you if you were considering help (for yourself, a close friend, or a close family member?)

Information type	Clinic sample (N=231)		Web sample (N=262)	
	Very important n (%)	Mean rating (95% CI)	Very important n (%)	Mean rating (95% CI)
Training of person providing treatment	185 (80.0)	6.8 (6.54-6.96)	207 (79.0)	6.6 (6.41-6.85)
Health care provider's experience in treatment these problems	192 (83.1)	6.8 (6.56-6.97)	218 (83.2)	6.8 (6.63-7.01)
Waiting period before starting treatment	164 (71.0)	6.3 (6.03-6.52)	220 (84.0)	**7.0 (6.76-7.14)**[a]
Where treatment will take place	129 (55.8)	5.6 (5.30-5.87)	168 (64.1)	5.9 (5.69-6.17)
Amount of time required to take treatment	141 (60.8)	5.8 (5.56-6.08)	176 (67.2)	6.0 (5.81-6.27)
Time of day appointment's scheduled	118 (51.1)	5.2 (4.88-5.47)	162 (61.8)	**5.8 (5.55-6.07)**[a]
Treatment option health care provider recommends and reasons why	176 (76.2)	6.4 (6.15-6.61)	218 (83.2)	**6.8 (6.63-7.01)**[a]

[a]Web sample and Clinic sample CIs do not overlap.

[b]Each source was rated on a 9-point rating scale with the anchors 0-2 (not important), 3-5 (moderately important), and 6-8 (very important).
