# Supplementary material for: Gathering Opinions on Depression Information Needs and Preferences: Samples and Opinions in Clinic Versus Web-Based Surveys
Source: JMIR Ment Health. 2017 Apr 24;4(2):e13. doi: 10.2196/mental.7231 (PMC5422653; doi:10.2196/mental.7231)
Supplement: Multimedia Appendix 4 [file mental_v4i2e13_app4.pdf]

Multimedia Appendix 4

Sociodemographic Characteristics of Respondents

Characteristic		No Honourarium (N=128)	Honourarium (N=149)	Statistical comparison
Mean age (SD[a])		40.4 (11.81)	40.0 (13.03)	t_{277} = .31, *P*=.76
Gender proportion, n (%)	Female	115 (89.8)	118 (79.2)	χ^2_1 = 5.8, *P*=.02[b]
	Male	13 (10.2)	31 (20.8)	
White, n (%)	Yes	115 (89.8)	124 (83.2)	χ^2_1 = 2.6, *P*=.11
	No	13 (10.2)	25 (16.8)	
Married, n (%)	Yes	69 (53.9)	72 (48.3)	χ^2_1 = .86, *P*=.35
	No	59 (46.1)	77 (51.7)	
Working full-time proportion, n (%)		61 (47.7)	86 (57.7)	χ^2_1 = 2.8, *P*=.094
Mean years education (SD)		14.5 (5.21)	13.7 (5.21)	t_{277} = 1.30, *P*=.20
Distress score (SD)		11.2 (6.50)	9.9 (5.81)	t_{277} = 1.65, *P*=.10
Received counseling for depression, n (% yes)		109 (85.2)	113 (75.8)	χ^2_1 = 3.8, *P*=.05
Counseling for depression would have been helpful but not received, n (% yes)		100 (78.1)	109 (73.2)	χ^2_1 = .92, *P*=.34
Received medication for depression, n (% yes)		99 (77.3)	101 (67.8)	χ^2_1 = 3.1, *P*=.08
Medication for depression would have been helpful but not received, n (% yes)		54 (42.2)	52 (34.9)	χ^2_1 = 1.6, *P*<.21

[a]SD: standard deviation.

[b]Significantly different means or proportions between the 2 samples, where *P*<.05.
