# Supplementary material for: Gathering Opinions on Depression Information Needs and Preferences: Samples and Opinions in Clinic Versus Web-Based Surveys
Source: JMIR Ment Health. 2017 Apr 24;4(2):e13. doi: 10.2196/mental.7231 (PMC5422653; doi:10.2196/mental.7231)
Supplement: Multimedia Appendix 5 [file mental_v4i2e13_app5.pdf]

Multimedia Appendix 5

Treatment Options: What information would be important to you if you were considering help (for yourself, a close friend, or a close family member?)

Information Type	No Honourarium (N=128)		Honourarium (N=149)	
	Very Important n (%)	Mean Rating (95% CI)	Very Important n (%)	Mean Rating (95% CI)
All available treatments	114 (89.1)	7.3 (7.02-7.47)	133 (89.3)	7.2 (7.02-7.46)
Available medication treatments	93 (72.7)	6.5 (6.12-6.8)	101 (67.8)	6.2 (5.91-6.57)
Available counseling or psychological treatments	117 (91.4)	7.3 (7.07-7.53)	130 (87.2)	7.2 (6.93-7.41)
Self-help treatment	99 (77.3)	6.7 (6.39-6.97)	116 (77.9)	6.6 (6.32-6.91)
Herbal remedies	51 (39.8)	4.5 (4.06-4.95)	64 (43.0)	4.8 (4.41-5.18)
Exercise	99 (77.3)	6.6 (6.25-6.87)	106 (71.1)	6.4 (6.08-6.72)
Bright light therapy	85 (66.4)	5.8 (5.46-6.19)	97 (65.1)	5.9 (5.51-6.21)
What you have to do as part of the treatment	111 (86.7)	7.2 (6.94-7.43)	131 (87.9)	7.0 (6.76-7.31)
Cost of treatment to you	101 (78.9)	6.9 (6.55-7.17)	118 (79)	6.7 (6.38-7.05)
Cost of treatment to healthcare system	32 (25.0)	3.9 (3.49-4.31)	39 (26.2)	3.7 (3.25-4.06)
Effectiveness of treatment	118 (92.2)	7.4 (7.15-7.59)	133 (89.3)	7.2 (6.96-7.45)
How treatment works	111 (86.7)	7.2 (6.92-7.41)	127 (85.2)	6.9 (6.66-7.18)
Goal or outcome of treatment	114 (89.1)	7.2 (7.02-7.47)	137 (92.0)	7.3 (7.09-7.52)
How long it takes for treatment to produce results	105 (82.0)	6.8 (6.56-7.10)	125 (83.9)	6.9 (6.65-7.19)
How long treatment continues	106 (82.8)	6.8 (6.54-7.12)	121 (81.2)	6.8 (6.47-7.02)
What happens when treatment stops	117 (91.4)	7.2 (6.97-7.44)	130 (87.3)	7.1 (6.88-7.40)
Common side effects of treatment	111 (86.7)	7.2 (6.99-7.47)	128 (85.9)	7.0 (6.78-7.29)
Uncommon but serious side effects of treatment	101 (78.9)	6.8 (6.47-7.04)	124 (83.2)	6.9 (6.59-7.15)
Advantages and disadvantages of treatment	117 (91.4)	7.3 (7.07-7.54)	131 (87.9)	7.0 (6.72-7.22)

[a]Each source was rated on a 9-point rating scale with the anchors 0-2 (not important), 3-5 (moderately important), and 6-8 (very important).
