# Supplementary material for: Gathering Opinions on Depression Information Needs and Preferences: Samples and Opinions in Clinic Versus Web-Based Surveys
Source: JMIR Ment Health. 2017 Apr 24;4(2):e13. doi: 10.2196/mental.7231 (PMC5422653; doi:10.2196/mental.7231)
Supplement: Multimedia Appendix 6 [file mental_v4i2e13_app6.pdf]

Multimedia Appendix 6

How likely would you be to talk to one of the following people for advice if you were having a serious problem with depression?

Source of Advice	No Honourarium (N=114)		Honourarium (N=149)	
	Very Likely n (%)	Mean Rating (95% CI)	Very Likely n (%)	Mean Rating (95% CI)
Romantic partner/spouse	72 (63.2)	5.8 (5.31-6.26)	89 (59.7)	5.5 (5.10-5.99)
Parent	27 (23.7)	3.2 (2.71-3.74)	40 (26.9)	3.6 (3.15-4.05)
Family member (not parent)	33 (29.0)	3.8 (3.30-4.30)	43 (28.9)	3.8 (3.39-4.21)
Friend	63 (55.3)	5.3 (4.81-5.68)	78 (52.4)	5.2 (4.78-5.56)
Phone-in counseling or health line	31 (27.2)	3.6 (3.14-4.12)	52 (34.9)	4.3 (3.87-4.64)
Counselor or therapist	82 (71.9)	6.6 (6.22-6.90)	124 (83.2)	6.7 (6.43-7.04)
Religious leader or community elder	18 (15.8)	1.9 (1.32-2.37)	25 (16.8)	2.3 (1.83-2.74)
Family doctor	75 (65.8)	6.0 (5.52-6.37)	107 (71.8)	6.2 (5.83-6.52)

[a]Each source was rated on a 9-point rating scale with the anchors 0-2 (not important), 3-5 (moderately important), and 6-8 (very important).
