# Supplementary material for: Gathering Opinions on Depression Information Needs and Preferences: Samples and Opinions in Clinic Versus Web-Based Surveys
Source: JMIR Ment Health. 2017 Apr 24;4(2):e13. doi: 10.2196/mental.7231 (PMC5422653; doi:10.2196/mental.7231)
Supplement: Multimedia Appendix 7 [file mental_v4i2e13_app7.pdf]

Multimedia Appendix 7

Preferred method of receiving information about services

Preferred Method	No Honourarium (N=114)		Honourarium (N=149)	
	Very Preferred n (%)	Mean Rating (95% CI)	Very Preferred n (%)	Mean Rating (95% CI)
Written form (information sheet)	67 (58.8)	6.0 (5.58-6.33)	97 (65.1)	6.1 (5.70-6.41)
Discussion with medical doctor	64 (56.1)	5.5 (5.16-5.88)	91 (61.1)	5.8 (5.46-6.11)
Discussion with counselor/therapist	88 (77.2)	6.3 (5.91-6.60)	106 (71.1)	6.2 (5.92-6.53)
Video on the Internet	31 (27.2)	4.1 (3.71-4.55)	49 (32.9)	4.3 (3.90-4.65)
Recommended website accessed from home	63 (55.3)	5.6 (5.24-5.95)	86 (57.7)	5.6 (5.30-5.96)

[a]Each source was rated on a 9-point rating scale with the anchors 0-2 (*not preferred*), 3-5 (*moderately preferred*), and 6-8 (*very preferred*).
