# Supplementary material for: Gathering Opinions on Depression Information Needs and Preferences: Samples and Opinions in Clinic Versus Web-Based Surveys
Source: JMIR Ment Health. 2017 Apr 24;4(2):e13. doi: 10.2196/mental.7231 (PMC5422653; doi:10.2196/mental.7231)
Supplement: Multimedia Appendix 8 [file mental_v4i2e13_app8.pdf]

Multimedia Appendix 8

How helpful would the following types of assistance be if you were having a problem with depression?

Type of assistance	No Honourarium (N=113)		Honourarium (N=149)	
	Very Helpful n (%)	Mean Rating (95% CI)	Very Helpful n (%)	Mean Rating (95% CI)
Recommended self-help book	44 (38.9)	4.8 (4.37-5.25)	69 (46.3)	5.0 (4.65-5.37)
Recommended self-help website	50 (44.2)	5.1 (4.68-5.50)	73 (49.0)	5.2 (4.82-5.51)
Telephone meetings with a counselor	50 (44.2)	4.8 (4.31-5.23)	81 (54.4)	5.2 (4.83-5.54)
In person meetings with a counselor	97 (85.8)	7.0 (6.67-7.26)	127 (85.2)	6.9 (6.61-7.18)
Educational meeting (about 2 hours with 20-30 people)	40 (35.4)	4.1 (3.63-4.62)	55 (36.9)	4.3 (3.87-4.72)
Educational workshop (about 6 hours with 20-30 people)	44 (38.9)	4.2 (3.67-4.72)	58 (38.9)	4.1 (3.67-4.53)
Internet discussion group led by a professional	28 (24.8)	3.7 (3.23-4.14)	36 (24.2)	3.7 (3.35-4.09)
Internet discussion group led by a person who has coped with the problem themselves	36 (31.9)	3.9 (3.38-4.33)	43 (28.9)	3.8 (3.38-4.16)
Medication recommended by your family doctor	60 (53.1)	5.5 (5.02-5.88)	86 (57.7)	5.4 (5.04-5.78)
Medication recommended by a specialist in psychiatry	74 (65.5)	6.0 (5.55-6.40)	97 (65.1)	5.8 (5.39-6.14)

[a]Each source was rated on a 9-point rating scale with the anchors 0-2 (not important), 3-5 (moderately important), and 6-8 (very important).
